# Calibration and functional analysis of three genetically encoded Cl^−^/pH sensors

**DOI:** 10.3389/fnmol.2013.00009

**Published:** 2013-04-18

**Authors:** M. Mukhtarov, L. Liguori, T. Waseem, F. Rocca, S. Buldakova, D. Arosio, P. Bregestovski

**Affiliations:** ^1^Brain Dynamics Institute, Inserm UMR1106, Aix-Marseille UniversityMarseille, France; ^2^Laboratory of Neurobiology, Institute of Fundamental Medicine and Biology, Kazan Federal UniversityKazan, Russia; ^3^Institute of Biophysics, National Research Council and FBKTrento, Italy; ^4^Institute of Biophysics and Cell Engineering, National Academy of SciencesMinsk, Belarus; ^5^Physics Department, University of TrentoTrento, Italy

**Keywords:** fluorescent biosensors, intracellular chloride, intracellular pH, non-invasive monitoring, optogenetics

## Abstract

Monitoring of the intracellular concentrations of Cl^−^ and H^+^ requires sensitive probes that allow reliable quantitative measurements without perturbation of cell functioning. For these purposes the most promising are genetically encoded fluorescent biosensors, which have become powerful tools for non-invasive intracellular monitoring of ions, molecules, and enzymatic activity. A ratiometric CFP/YFP-based construct with a relatively good sensitivity to Cl^−^ has been developed (Markova et al., 2008; Waseem et al., 2010). Recently, a combined Cl^−^/pH sensor (ClopHensor) opened the way for simultaneous ratiometric measurement of these two ions (Arosio et al., 2010). ClopHensor was obtained by fusion of a red-fluorescent protein (DsRed-monomer) to the E^2^GFP variant that contains a specific Cl^−^-binding site. This construct possesses p*K*_*a*_ = 6.8 for H^+^ and *K*_*d*_ in the 40–50 mM range for Cl^−^ at physiological pH (~7.3). As in the majority of cell types the intracellular Cl^−^ concentration ([Cl^−^]_*i*_) is about 10 mM, the development of sensors with higher sensitivity is highly desirable. Here, we report the intracellular calibration and functional characterization of ClopHensor and its two derivatives: the membrane targeting PalmPalm-ClopHensor and the H148G/V224L mutant with improved Cl^−^ affinity, reduced pH dependence, and p*K*_*a*_ shifted to more alkaline values. For functional analysis, constructs were expressed in CHO cells and [Cl^−^]_*i*_ was changed by using pipettes with different Cl^−^ concentrations during whole-cell recordings. *K*_*d*_ values for Cl^−^ measured at 33°C and pH ~7.3 were, respectively, 39, 47, and 21 mM for ClopHensor, PalmPalm-ClopHensor, and the H148G/V224L mutant. PalmPalm-ClopHensor resolved responses to activation of Cl^−^-selective glycine receptor (GlyR) channels better than did ClopHensor. Our observations indicate that these different ClopHensor constructs are promising tools for non-invasive measurement of [Cl^−^]_*i*_ in various living cells.

## Introduction

Beginning with the pioneer studies by Tsien and co-authors on measurements of intracellular calcium concentration in intact lymphocytes (Tsien et al., 1982), non-invasive monitoring of different intracellular ions (Ca^2+^, Mg^2+^, Cl^−^, and H^+^) has became a powerful direction of research for functional analysis of neurons and other cell types under normal and pathophysiological conditions.

Among several methods proposed for monitoring intracellular Cl^−^ concentration ([Cl^−^]_*i*_), the most promising is the use of genetically encoded Cl^−^-sensitive probes (Bregestovski et al., 2009; Mancuso et al., 2011). The first generation of probes named “Clomeleon” (Kuner and Augustine, 2000) and “Cl-Sensor” (Markova et al., 2008) was based on the Cl^−^-sensitive yellow-fluorescent protein (YFP) linked with Cl^−^-insensitive cyan-fluorescent protein (CFP), which was the reference fluorescence molecule. These indicators have opened the way for non-invasive monitoring and ratiometric measurement of [Cl^−^]_*i*_ in different cell types *in vitro* (Pellegrino et al., 2011; Bertollini et al., 2012; Friedel et al., 2013). Expressed in neurons of transgenic mice, they have allowed imaging of Cl^−^ dynamics in inhibitory circuits of different brain areas, including hippocampus, cerebellum, and deep cerebellar nuclei (Berglund et al., 2008, 2011), as well as in intact hippocampus (Dzhala et al., 2012) and a dorsal root ganglia preparation (Batti et al., 2013). Producing a construct consisting of a glycine receptor (GlyR) with Cl-Sensor incorporated into the long cytoplasmic domain (BioSensor-GlyR) provided a tool for non-invasive monitoring activity of these Cl^−^-selective receptor-operated channels (Mukhtarov et al., 2008).

The recently proposed combined Cl^−^/pH sensor (ClopHensor) opened the way for simultaneous ratiometric measurement of these two ions (Arosio et al., 2010). ClopHensor is obtained by fusion of a red-fluorescent protein (DsRed-monomer) to the E^2^GFP variant that contains a specific Cl^−^-binding site. This construct is particularly promising as it allows simultaneous monitoring of [Cl^−^]_*i*_ and intracellular pH (pH_*i*_) in the same cells (Arosio et al., 2010; Bregestovski and Arosio, 2011; Raimondo et al., 2012). At physiological pH (~7.3), ClopHensor possesses p*K*_*a*_ = 6.8 for H^+^ and *K*_*d*_ in the 40–50 mM range for Cl^−^. As in the majority of cell types the intracellular Cl^−^ concentration is about 10 mM (Khirug et al., 2008; Tyzio et al., 2008; Bregestovski et al., 2009), development of sensors with higher sensitivity is necessary.

Here, we present calibration and functional analysis of ClopHensor and two of its derivatives: (i) PalmPalm-ClopHensor, which should have preferable membrane targeting and thus allow near-membrane measurement of [Cl^−^]_*i*_ and pH_*i*_; and (ii) ClopHensor with mutations H148G and V224L aimed to increase the affinity of this probe for Cl^−^ and change the pH-sensing properties. It has been previously demonstrated that the V224L mutation increases the affinity for Cl^−^ by an order of magnitude (Arosio et al., 2011), while the H148G mutation increases the p*K*_*a*_ value by 1 pH unit in YFP (Elsliger et al., 1999) as well as in GFP (Hanson et al., 2002). Here, we combined the H148G and the V224L mutations in E^2^GFP in order to: (1) shift the *K*_*d*_ for Cl to the lower mM range, and (2) increase the p*K*_*a*_ of the GFP sensing element to more alkaline values.

For these three constructs, calibrations determining their sensitivity to Cl^−^ and H^+^ in living cells were obtained. Also presented are the distributions of these probes in cells and simultaneous recording of changes in [Cl^−^]_*i*_ and pH_*i*_ during activation of Cl^−^-selective GlyR channels.

## Materials and methods

### Producing and cloning of ClopHensor variants

The ClopHensor, E^2^GFP-DsRedm, construct was mutated at two residues, H148G and V224L, of the GFP domain. Two sequential site-directed mutagenesis were performed using QuickChange II XL Site-Directed Mutagenesis Kit (Stratagene) following the manufacturer's protocol. Complementary primers were synthesized by Sigma-Aldrich with the following sequences: H148G-fw GAGTACAACTACAACAGCGG CAACGTCTATATCATGG; H148G-rv CCATGATATAGACGTTGCCGCTGTTGT AGTTGTACTC; V224L-fw CTGCTGGAGTTCCTGAACGCCGCCG; V224L-rv CGGCGGCGTTCAGGAACTCCAGCAG and were used to amplify the entire plasmid in a PCR reaction using high-fidelity polymerase. To eliminate template, the PCR reaction was digested with Dpn1. The amplified mutated DNA was purified using Wizard SV Gel and the PCR Clean-up System kit (Promega), and transformed into *Escherichia coli* XL10-Gold ultracompetent cells (Novagen), which were then grown overnight on LB plates supplemented with 50 mg/l ampicillin at 37°C.

Four positive colonies were picked and grown overnight in 3 ml of LB-ampicillin at 37°C under shaking for mini prep DNA extraction (Wizard®*Plus* SV Minipreps DNA Purification; Promega). All the constructs were verified by sequencing the entire insert. Finally, plasmids used for transfection were prepared using the QIAGEN Plasmid *Plus* Maxi kit.

### Expression and purification of the H148G/V224L mutant

The recombinant GFP variant was expressed as Strep-tagged protein in *Escherichia coli* BL21 (Novagen) and harvested 20 h after induction with 1 mM IPTG at 30°C. Purification by affinity was carried out using Strep-Tactin Superflow 5-ml cartridges (IBA, GmbH, Germany), following the manufacturer's instructions, at 4°C in an AKTA Basic10 FPLC system (GE Healthcare Europe, Milan, Italy) with continuous monitoring of optical densities at 280 nm. The use of Cl^−^-free buffers in the final purification step ensured the complete removal of Cl^−^ from the preparation. Diethanolamine (DEA; 20 mM) in 50 mM K_2_SO_4_ adjusted to pH 8.5 was supplemented with a cocktail of protease inhibitors (Roche). Lysis was performed with an Ultrasonic Processor (Cole Parmer) (10 cycles of 30 s, output 6W, and 10 s cooling). FPLC was performed on an AKTA Basic10 FPLC system (GE Healthcare) using a Strep-Tactin Superflow 5 ml column (IBA Technology). Filtered lysate was loaded without a loop, and the flow rate was set at 3 ml/min. Elution of bound strep-tagged GFP was obtained in 75% washing buffer 2.5 mM desthiobiotin. Protein Concentration was determined by BCA™ Protein Assay (Pierce Protein Biology Products, USA).

### Cell culture preparation

For fluorescence analysis of [Cl^−^]_*i*_ and [H^+^]_*i*_, and also for immunocytochemistry and electrophysiology, ClopHensor constructs and human α1 GlyR subunits were expressed in CHO cells and in neurons of primary culture by means of Lipofectamin transfection.

Chinese hamster ovary (CHO-K1) cells were obtained from the American Type Tissue Culture Collection (ATCC, Molsheim, France) and maintained as previously described (Medina et al., 2000; Waseem et al., 2010). One day before the transfection, cells were plated onto coverslips (12–14 mm in diameter), which were placed inside 35-mm cell culture dishes with 2 ml of medium. CHO-K1 cells were transfected with approximately 1 μg/1μl cDNA of constructs, using the Lipofectamine 2000 transfection protocol (Life Technology, USA). Three hours after the initial exposure of the cells to the cDNAs, a fresh cDNA-containing solution replaced the old one.

Neurons of hippocampal culture from 18-day rat embryos were dissociated using trypsin and plated at a density of 70,000 cells cm^−2^ in minimal essential medium (MEM) supplemented with 10% NU serum (BD Biosciences, Le Pont de Claix, France), 0.45% glucose, 1 mM sodium pyruvate, 2 mM glutamine, and 10 IU ml^−1^ penicillin–streptomycin as previously described (Buerli et al., 2007). On days 7, 10, and 13 of culture incubation, half of the medium was changed to MEM with 2% B27 supplement (Invitrogen).

Transfections of neuronal cultures at 7–10 days *in vitro* (DIV) were performed as described previously (Buerli et al., 2007; Pellegrino et al., 2011). Cells were used in experiments 2–5 days after transfection.

### Intracellular Cl^−^ calibration of ClopHensor variants

For Cl^−^ calibration we used whole-cell recordings with different Cl^−^ concentrations in recording pipettes. Whole-cell patch-clamp recordings on CHO cells were conducted 24–48 h after transfection, using an EPC-9 amplifier (HEKA Elektronik, Germany) at a holding potential −20 or −30 mV and at a temperature of 32–33°C. Cells were bathed in a solution containing (mM): NaCl 126; KCl 3.5; CaCl_2_ 2; MgCl_2_ 1.3; NaH_2_PO_4_ 1.2; NaHCO_3_ 25; and D-glucose 10; equilibrated at pH 7.4 with 95% O_2_ and 5% CO_2_; 320 mOsm. The patch pipette solution contained (mM): KCl (0–135) or K-gluconate (0–135); MgCl_2_ 2; MgATP 2; HEPES/KOH 10; and BAPTA 1; pH 7.3, 300 mOsm. A combination of K-gluconate and KCl at a constant K^+^ concentration of 135 mM were used for Cl^−^ calibration of ClopHensor constructs with six different Cl^−^ concentrations in the pipette solution ([Cl^−^]_*p*_): 4, 10, 20, 60, 100, and 135 mM. Calibration curves were obtained by recording from 5–7 cells for each [Cl^−^]_*p*_. The effectiveness of the cell dialysis and [Cl^−^]_*i*_ established after whole-cell penetration were checked by measurement of the reversal potential for glycine-induced currents. Glycine was applied locally using Picospritzer II (General Valve Corporation, USA) with a pipette positioned close to the soma of the recorded cell at different holding potentials.

All reagents were obtained from Sigma unless otherwise specified.

### Intracellular pH calibration of ClopHensor variants

For pH calibration b-escin permabilization method was used (Waseem et al., 2010). In more detail, a range of HEPES-based extracellular solutions (150 mM K-Gluconate, 20 mM HEPES, and 10 mM D-glucose) with different pH values was created by adding HEPES powder for acidification of the solution or 1 M NaOH for alkalization.

To increase the permeability of the cell membrane to ions, 80 μM β-escin (Sigma, St Louis, USA) was added to the bath solution. β-escin was dissolved in water and prepared freshly for each experiment. This suspension was stable for about 2 h. The coverslip with cultured cells was placed into the recording chamber and superfused with escin-containing bath solution until cells become swollen, indicating the dissipation of ion gradients and coupled membrane potential in treated cells. The perfusing solution was then switched to the escin-free bath solution in order to avoid lysis of the cells. Thereafter, the fluorescence responses of ClopHensor constructs corresponding to specified H^+^ concentrations inside the cell were registered.

### Real-time fluorescence imaging

Fluorescence images were acquired using a customized digital imaging microscope. Excitation of cells at various wavelengths was achieved using a Polychrome V monochromator (Till Photonics, Germany). Light intensity was attenuated using neutral density filters. Emission wavelengths were controlled using a Lambda 10-3 controller (Sutter Instrument Company, USA). Fluorescence was visualized using an upright microscope Axioskop (Zeiss, Germany) equipped with a 60× water-immersion objective (n.a. 0.9; LumPlanFL, Olympus, USA). Fluorescent emitted light passed to a 16-bit digital camera Andor iXon EM+ (Andor Technology PLC, Northern Ireland). Images were acquired on a computer via a DMA serial transfer. All peripheral hardware control, image acquisition and image processing were achieved using Andor iQ software (Andor Technology PLC). The average fluorescence intensity of each region of interest (ROI) was measured.

Cells expressing ClopHensor variants were excited at three wavelengths: 458 and 488 nm for Cl^−^/pH-sensitive E^2^GFP excitation, and 545 nm for excitation of DsRed-monomer. Fluorescent signals were recorded using a dual-band GFP/DsRed 493/574 dichroic mirror (Semrock Inc., USA) and two emission filters: 535 ± 15 nm for E^2^GFP emission and 632 ± 30 nm for emission of DsRed-monomer (both Chroma Technology Corporation, USA). The emission filters were mounted into the Lambda 10^−3^ Filter wheel (Sutter Instruments Company, Novato, USA).

The duration of excitation at each wavelength was usually 20 ms. The sampling interval was 5 s for the slow [Cl^−^]_*i*_ changes during the transition of the recorded cell in whole-cell configuration and was switched to 1 s for the fast [Cl^−^]_*i*_ transients in response to glycine application after establishing [Cl^−^]_*i*_/[Cl^−^]_*p*_ equilibrium.

### Immunocytochemistry

For immunodetection of constructs in CHO cells or hippocampal neurons, cells in culture on coverslips expressing ClopHensor or it derivatives were fixed in PFA 4% (wt/vol) in 0.1 M phosphate buffer (PBS), pH 7.4, at room temperature for 15 min. After fixation, the cells were rinsed three times in PBS. In each experimental procedure, control and experimental cells were processed together to eliminate potential bias due to inherent variations in the intensity of the immunohistochemical labeling. For further staining, neurons were pre-incubated in 4% (vol/vol) normal goat serum in PBS containing 0.5% Triton X-100 (vol/vol) for 1 h at room temperature to block secondary-antibody binding sites. Coverslips with neurons expressing ClopHensor were then incubated overnight at 4°C with rabbit anti-GFP polyclonal antibody (Invitrogen) diluted 1:4000 in PBS with 4% normal goat serum and mouse monoclonal anti-MAP2 antibodies (1:2000). Incubations with the primary antibodies were performed at room temperature and slow shaking for 1 h, then overnight at 4°C. Samples were rinsed three times in PBS. As secondary antibodies, either a Cy3-conjugated anti-rabbit IgG for detection of GFP or a Cy5-conjugated anti-mouse IgG for detection MAP2 (all 1:1500; Molecular Probes, Invitrogen) were used. Samples were then rinsed twice in PBS with one additional washing in PBS containing Hoechst 33342 (10 mg/ml; Sigma-Aldrich). Coverslips were mounted using Fluoromount™ (Sigma-Aldrich, St Louis, MO, USA).

Images were acquired using a Leica SP5C confocal microscope using 40–60× oil-immersion objectives. Micrographs shown here are digital composites of *Z*-series scans of 5–15 optical sections through a depth of 1–6 μm. Final images were constructed with ImageJ software.

### *In vitro* analysis

*In vitro* characterization of H^+^ and Cl^−^-binding properties was performed by analysing fluorescence spectra variations with a multimodal plate reader (EnSpire; PerkinElmer) in 96-well plates. Acetic acid buffer (50 mM) was used to adjust the solution pH to around 5.2, 100 mM PBS was used for the pH range 5.2–8.0 and 20 mM DEA buffer was used for the pH range 8.0–8.5. Temperature was kept constant at 20 ± 0.5°C and fluorescence spectra were acquired with 1-nm steps. Protein concentration was always kept constant at about 1 μM throughout every pH and Cl titration.

The proton dissociation constant (p*K*_*a*_) was obtained by fitting fluorescence data with the equation:

$$\text{F}=\frac{A_{1}+A_{2}10^{\left(\text{p}K_{a}\;-\text{pH}\right)}}{1+10^{\left(\text{p}K_{a}-\text{pH}\right)}}$$

which describes the effect of environmental proton concentration (pH) on chromophore fluorescence (*F*).

The chloride dissociation constant (*K*^*Cl*^_*d*_) was obtained by fitting data with a 1:1 Langmuir binding model (Arosio et al., 2007):

$$S=\frac{S_{0}+S_{1}\cdot \left[Cl\right]/K_{d}^{\text{Cl}}}{1+\left[Cl\right]/K_{d}^{\text{Cl}}}.$$

## Results

### Description of the constructs and *in vitro* analysis

**Figure 1:**
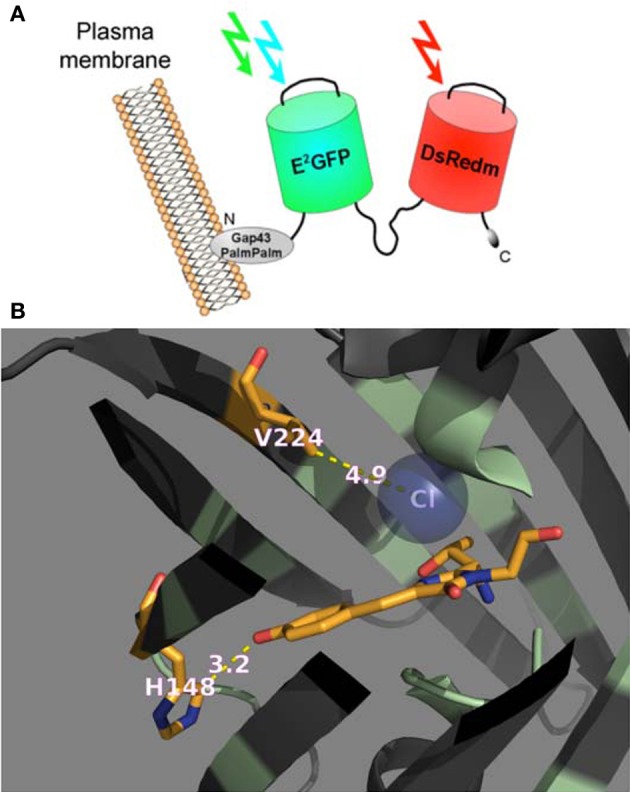
**Genetically encoded Cl^−^/pH-sensors used in this study. (A)** Schematic representation of PalmPalm-ClopHensor. **(B)** A cartoon representing the specific Cl^−^-binding site in the newly developed H148G/V224L/T203Y variant of E^2^GFP. The residues in positions 148 and 224 as well as the chromophore are shown as stick models with the atoms colored as follow: C, orange; N, dark blue, and O, red. The Cl^−^ ion is shown as a sphere model in transparent blue.

In this study we performed calibration and functional characterization of the original ClopHensor and doubly palmitoylated membrane-targeted constructs (Arosio et al., 2010), as well as a new ClopHensor variant with mutations designed to increase Cl^−^ affinity and shift p*K*_*a*_ to more alkaline values (Figure 1B).

ClopHensor was designed by linking two fluorescent proteins, E^2^GFP? EGFP-T203Y and DsRed-monomer by means of a flexible 20-amino-acid spacer. To target the sensor to the inner interface of the plasma membrane, we inserted the N-terminal 20 residues of GAP-43, which contain two palmitoylation sites, at the ClopHensor N-terminus (Figure 1A). DsRed fluorescence, which is not affected by pH or Cl^−^ changes, was shown to provide an excellent normalization signal leading to ratiometric Cl^−^ sensing, free from the influence of sensor concentration in living neurons.

**Figure 2:**
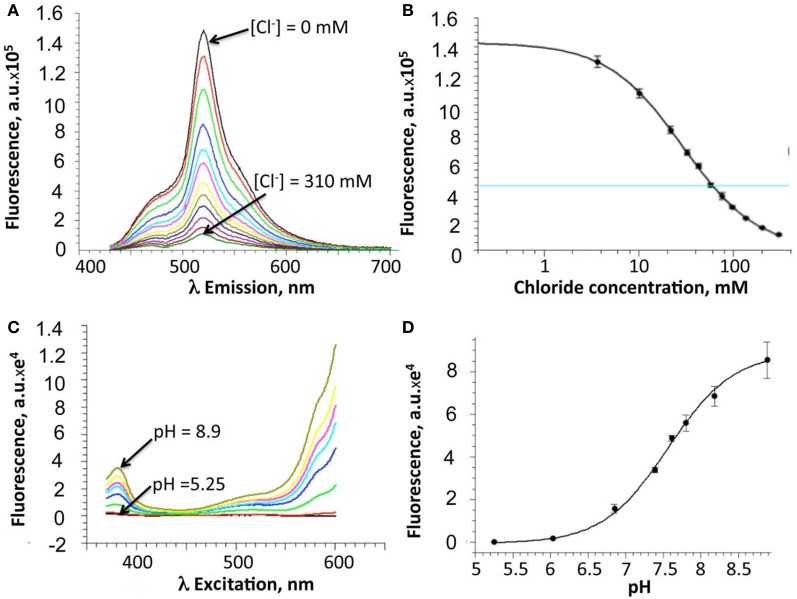
***In vitro* properties of the H148G/V224L ClopHensor mutant. (A)** Emission spectra at pH 7.0 for excitation at 415 nm, at increasing Cl^−^ concentrations from 0 mM (*top spectrum*) to 310 mM (*bottom spectrum*). Note the reduction in fluorescence intensity due to Cl^−^ binding-site quenching. **(B)** Dependence on Cl^−^ concentration of the fluorescence intensity recorded at 520 nm. **(C)** Excitation spectra, at different pH values, for emission at 520 nm, in buffer without chloride. *No isosbestic point is present*. **(D)** pH dependence of the fluorescence intensity recorded at 485 nm. For **(B)** and **(D)**, data are represented as mean ± SD from 3 independent measurements.

Following purification of the E^2^GFP-H148G-V224L mutant, Cl^−^-binding properties of the recombinant protein were investigated *in vitro* by measuring fluorescence spectra at constant pH and increasing Cl^−^ concentration, from 0 to 310 mM (Figures 2A,B). Because of the cooperative interaction between Cl^−^ and H^+^ binding, Cl^−^ titrations were measured at various pH values (from 5.25 to 8.9) and *K*_*d*_ values were analyzed with an infinite cooperative model (Arosio et al., 2007; Bregestovski and Arosio, 2011) with two interacting binding sites, one for Cl^−^ and one for H^+^:

$$K_{d}=\frac{K_{d}^{0}\cdot \left(1+10^{\left(\text{p}K_{a}-\text{pH}\right)}\right)}{10^{\left(\text{p}K_{a}-\text{pH}\right)}}$$

containing two fitting parameters for the probe: p*K*_*a*_ and *K*^0^_*d*_, the Cl^−^ dissociation constant for the fully protonated form (present at pH ≤ 5).

The proton binding equilibrium was studied and the p*K*_*a*_ value was determined from spectra acquired at different pH buffer conditions, ranging from 5.25 to 8.9, in the absence of halogens (Figures 2C,D).

Precisely, the Cl^−^ and H^+^ binding thermodynamic parameters for H148G/V224L were found to be p*K*_*a*_ = 7.9 ± 0.05 (best-fit ± SD) and *K*^0^_*d*_ = 19 ± 1.5 mM.

**Figure 3:**
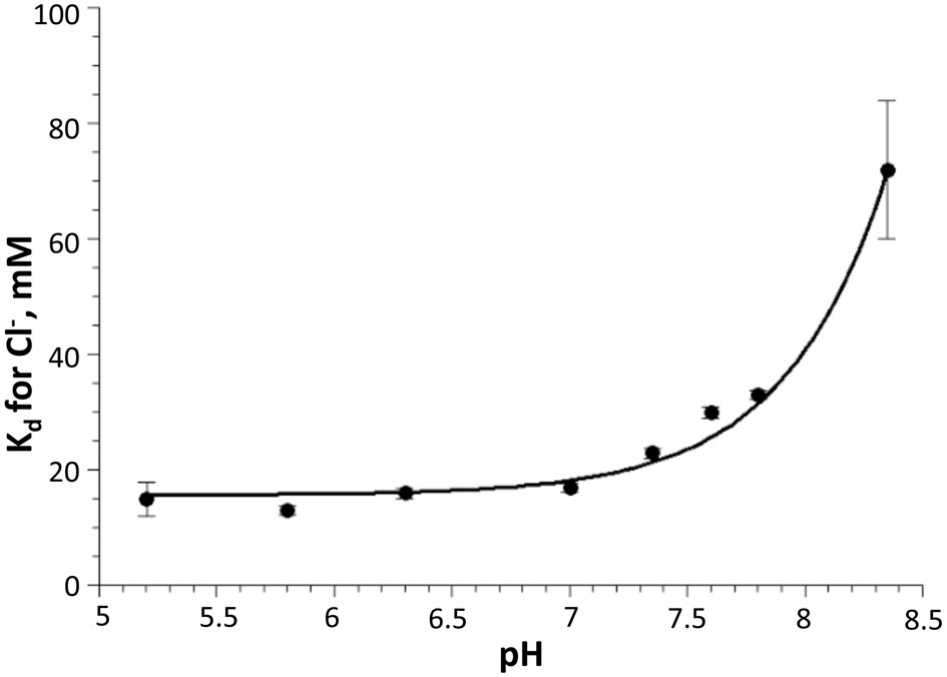
**Dependency of Cl^−^ dissociation constant *K*_*d*_ on pH for H148G/V224L mutant.** Note the relatively small variations of *K*_*d*_ in the physiological pH range (7.0–7.5). Data are represented as mean ± SD from 3 independent measurements.

Overall, *in vitro* analysis at various pH and Cl^−^ conditions revealed that the H148G/V224L mutant is suited for Cl^−^ concentration measurements with reduced pH dependence in the physiological range. Indeed, in the pH range between 6.8 and 7.8 *K*_*d*_ changes were between 18 and 30 mM (Figure 3).

### Distribution of the constructs in cells

**Figure 4:**
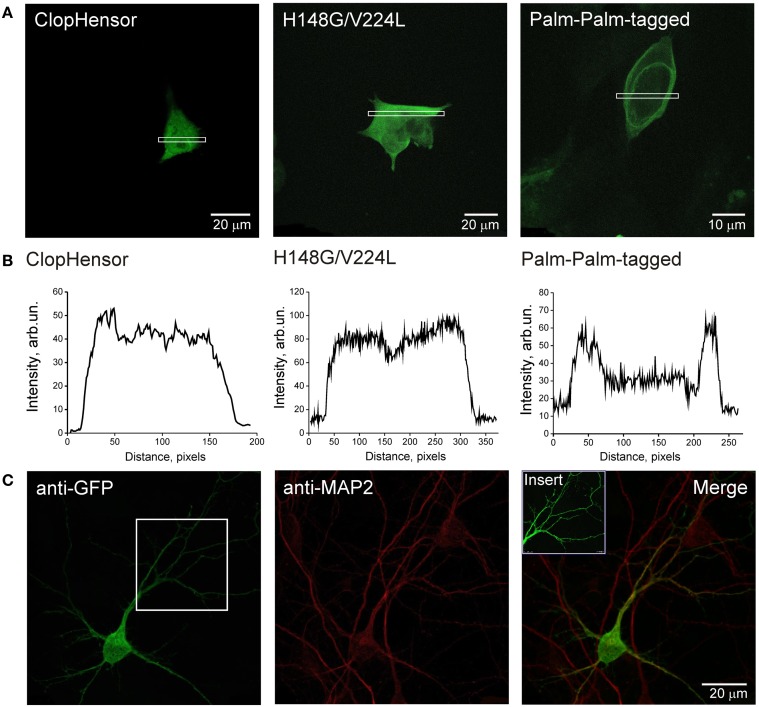
**Expression of ClopHensor and its derivatives in CHO cells and in hippocampal neurons. (A)** Confocal micrographs of CHO cells expressing three genetically encoded probes and **(B)** illustrative profiles of signal intensity distribution within the corresponding selected area. Cells were transfected with ClopHensor (*left*), H148G/V224L mutant (*middle*), and PalmPalm-ClopHensor (*right*). Note the difference in distribution patterns being predominantly cytoplasmic for both the ClopHensor and H148G/V224L mutant and preferential membrane localization for PalmPalm-ClopHensor that is additionally confirmed by profiles of fluorescent signal distribution. **(C)** Confocal micrographs of ClopHensor expressed in a hippocampal neuron (DIV14). The probe spread well throughout the cell body and processes, thus providing a suitable tool for [Cl^−^]_*i*_ and pH_*i*_ monitoring in different regions of neurons.

To determine the intracellular distribution of ClopHensor constructs, we transiently expressed them in CHO cells and in neurons of dissociated hippocampal culture (see Materials and Methods). Expression was already observable 12 h after transfection and remained at high levels for at least 4 days. The fluorescence of the ClopHensor was distributed homogeneously throughout CHO cells (Figures 4A,B, *left*) and in neurons (Figure 4C). The fluorescence was bright with no preferential staining of membrane or intracellular organelles, as illustrated by the fluorescence profile (Figure 4B, *left*).

Distribution of the H148G/V224L mutant was also cytoplasmic, similar to those for ClopHensor (Figures 4A,B, *middle*). In contrast, the PalmPalm-ClopHensor typically showed near-membrane distribution; however, a small fraction of the probe could still be observed in the cytoplasm (Figures 4A,B, *right*).

These observations indicate that ClopHensor and H148G/V224L mutants exhibit cytoplasmic intracellular distributions while the PalmPalm-ClopHensor construct is typically localized to the plasma membrane and the Golgi region, as it was shown previously (McCabe and Berthiaume, 1999).

### Intracellular calibration of the constructs

#### Calibration for Cl^−^

**Figure 5:**
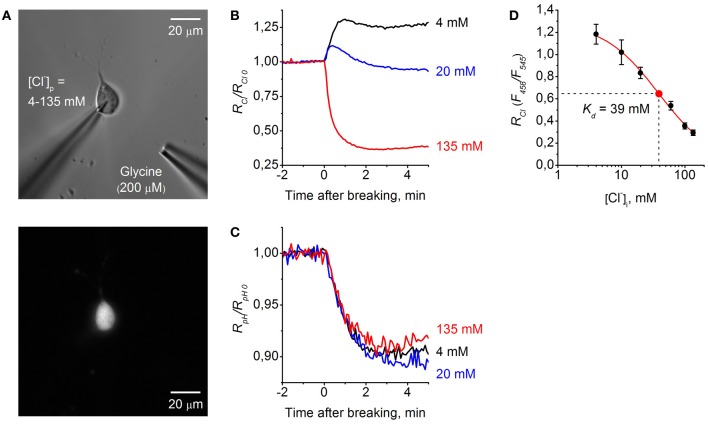
**Calibration of ClopHensor for Cl^−^. (A)** Micrographs of CHO cell co-transfected by ClopHensor and GlyR taken with white light (*top*) and 545 nm excitation (*bottom*). Note the patch pipette on the cell and puff pipette for glycine application at about 50 μm from the recorded cell. **(B)** Relative changes in *R*_*Cl*_ (*F*_458_/*F*_545_) measured in three cells during whole-cell recordings with different [Cl^−^]_*p*_: 4 mM (*black trace*), 20 mM (*blue trace*), and 135 mM (*red trace*). Each trace represents data from a single cell. Time = 0 corresponds to the moment the membrane breaks to give whole-cell mode. **(C)** Relative changes in *R*_*pH*_ (*F*_488_/*F*_458_) recorded simultaneously in the same cells as in **(B)** before and after cells transition into the whole-cell configuration. **(D)** Calibration curve for ClopHensor obtained by recording of *R*_*Cl*_ at six different [Cl^−^]_*p*_: 4, 10, 20, 60, 100, and 135 mM. *K*_*d*_ = 38.9 ± 6.5 mM (mean ± s.e.m.). Data from 5–7 cells for each [Cl^−^]_*p*_ are presented. Note the approximately four-fold change in *R*_*Cl*_ when [Cl^−^]_*i*_ is adjusted from 4 to 135 mM.

In order to evaluate the dynamic range and sensitivity of constructs to ions, we co-expressed them in CHO cells with Cl^−^-selective GlyR channels and performed simultaneous monitoring of whole-cell currents and fluorescent signals. Whole-cell recordings were performed with different Cl^−^ concentrations in the pipette solution ([Cl^−^]_*p*_ = 4–135 mM). To activate GlyR channels, glycine was applied using pressure pulses. For this, a pipette containing 200 μM glycine dissolved in extracellular solution was advanced to within 30–50 μm of the recorded cell (Figure 5A).

For ratiometric monitoring, following the approach presented in Arosio et al. (2010) we used the ratio *R*_*Cl*_ = *F*_458_/*F*_545_ for measurement of intracellular Cl^−^ concentration ([Cl^−^]_*i*_), and for calculating intracellular pH values (pH_*i*_) the ratio *R*_*pH*_ = *F*_488_/*F*_458_ was used.

Figure 5B illustrates relative changes in [Cl^−^]_*i*_ during whole-cell recordings from three cells, using pipettes with solutions containing different Cl^−^ concentrations. The graph represents changes in *R*_*Cl*_/*R*_*Cl*_0__ with time, where *R*_*Cl*_0__ corresponds to [Cl^−^]_*i*_ in the cell-attached mode, i.e., to the native concentration of Cl^−^ in the cytoplasm of CHO cells. Obtaining the whole-cell configuration by rupturing the membrane with a pipette containing 135 mM Cl^−^ resulted in a strong decrease in *R*_*Cl*_/*R*_*Cl*_0__, corresponding to elevation of [Cl^−^]_*i*_. In contrast, on rupture of the membrane with the pipette containing 4 mM Cl^−^ an increase in *R*_*Cl*_/*R*_*Cl*_0__ was observed. Transition to the whole-cell configuration with a pipette containing 20 mM Cl^−^ produced a short transient, increasing with stabilization of *R*_*Cl*_/*R*_*Cl*_0__ at a level close to those in the cell-attached configuration. This reflects the fact that in the recorded cell the native value of [Cl^−^]_*i*_ in cytoplasm is close to the [Cl^−^]_*p*_ of 20 mM.

Simultaneous monitoring of relative pH changes (*R*_*pH*_/*R*_*pH*_0__) showed that after transition to the whole-cell configuration pH_*i*_ similarly decreased for all three cells recorded with different [Cl^−^]_*p*_ but with identical pH (Figure 5C).

Calibration curves for Cl^−^ obtained from six different [Cl^−^]_*p*_ in physiological conditions were best fitted by a Logistic Dose–Response Sigmoidal Fit using the OriginPro 8.5 program with the formula:

$$R_{\text{C}l}=A2+\frac{A1-A2}{1+{\left(\frac{[Cl^{-}]i}{K_{d}}\right)}^{p}}$$

where *R*_*Cl*_ is the fluorescence ratio for Cl^−^ (*F*_458_/*F*_545_), *K*_*d*_ is the dissociation constant for Cl^−^binding, A1 and A2 are, respectively, the minimum and maximum asymptotic values of *R*_*Cl*_, and *p* is the power value.

From this formula we obtained the equation for [Cl^−^]_*i*_ values recalculation:

$${\left[Cl^{-}\right]}_{i}=K_{d}\cdot {\left(\frac{A1-A2}{R_{\text{Cl}}-A2}-1\right)}^{\frac{1}{p}}$$

For ClopHensor the values of constants obtained from fitting the curve were the following: *K*_*d*_ = 38.9 ± 6.5 mM, A1 = 1.29, A2 = 0.01, and *p* = 1 (Figure 5D).

**Figure 6:**
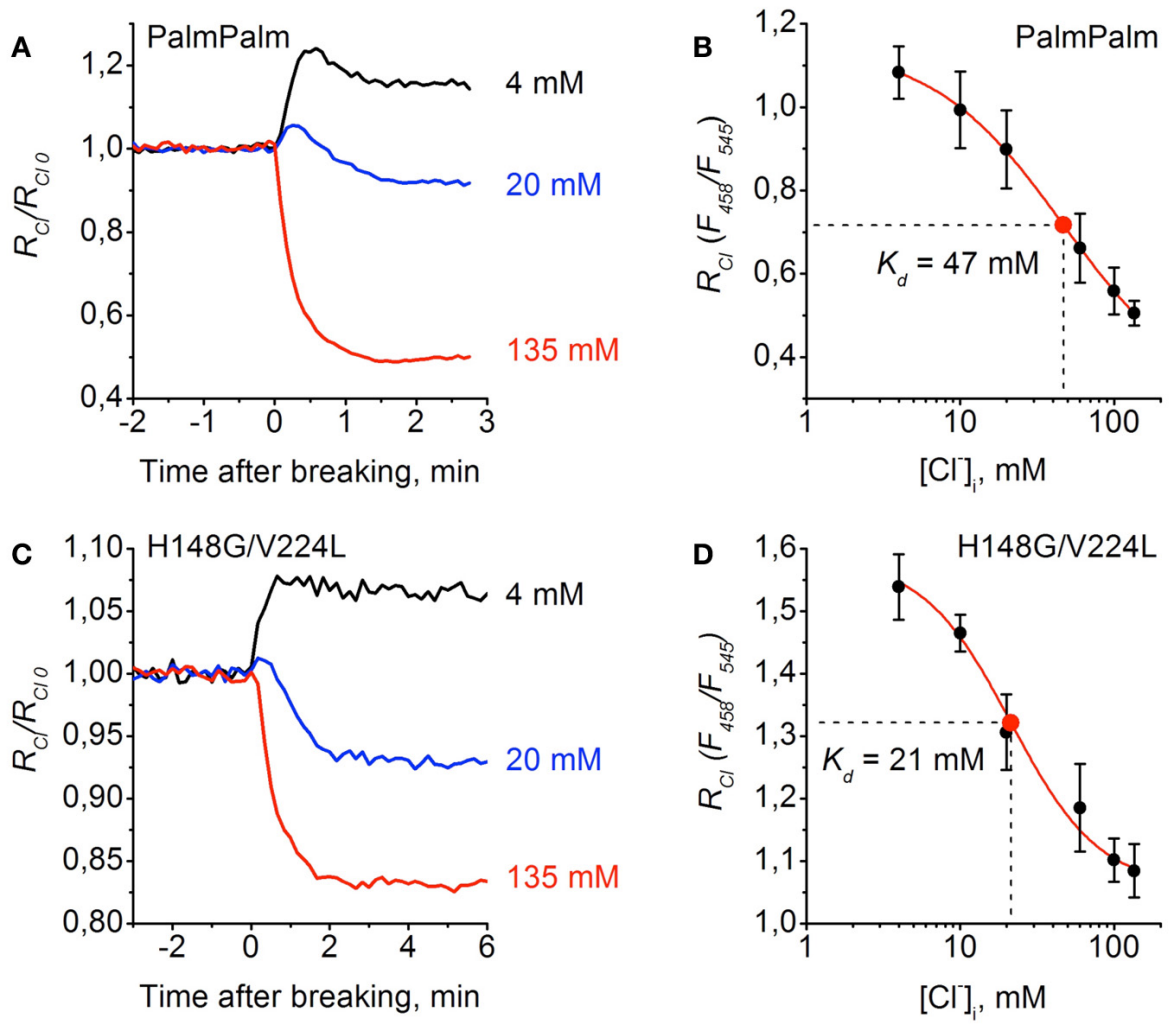
**Calibration of PalmPalm-ClopHensor and H148G/V224L mutant for Cl^−^. (A)** and **(C)** Relative changes in *R*_*Cl*_ (*F*_458_/*F*_545_) of PalmPalm-ClopHensor **(A)** and the H148G/V224L mutant **(C)** measured in three cells during whole-cell recordings with different [Cl^−^]_*p*_: 4 mM (*black trace*), 20 mM (*blue trace*), and 135 mM (*red trace*). Each trace represents the data from a single cell. Time = 0 corresponds to the moment the membrane breaks to give whole-cell mode. **(B)** and **(D)** Calibration curve for PalmPalm-ClopHensor **(B)** and H148G/V224L mutant **(D)** obtained by recording *R*_*Cl*_ at six different [Cl^−^]_*p*_: 4, 10, 20, 60, 100, and 135 mM. For PalmPalm-ClopHensor *K*_*d*_ = 46.8 ± 3.8 mM and for H148G/V224L mutant *K*_*d*_ = 21.4 ± 4.8 mM. Data from 5–7 cells for each [Cl^−^]_*p*_ are presented. Note the approximately two-fold changes in *R*_*Cl*_ for PalmPalm-ClopHensor when [Cl^−^]_*i*_ changed from 4 to 135 mM and the much smaller dynamic range of *R*_*Cl*_ for H148G/V224L mutant.

Similar analyses were performed for PalmPalm-ClopHensor and H148G/V224L mutants (Figure 6). They demonstrated that changes in *R*_*Cl*_/*R*_*Cl*_0__ observed at transitions from cell-attached to whole-cell configuration in cells expressing PalmPalm-ClopHensor were similar to those for ClopHensor (Figure 6A). From the calibration curve for PalmPalm-ClopHensor the following fitting parameters were obtained: *K*_*d*_ = 46.8 ± 3.8 mM, A1 = 1.15, A2 = 0.29, and *p* = 1.02 (Figure 6B). While this value of *K*_*d*_ was higher than for ClopHensor, the difference was non-significant (*P* > 0.05, *t*-Student).

For cells expressing the H148G/V224L mutant, *R*_*Cl*_/*R*_*Cl*_0__ when recording with the pipette containing 20 mM of Cl^−^ was almost midway between values recorded with pipettes containing 135 and 4 mM (Figure 6C), suggesting higher sensitivity of the construct to Cl^−^. This was confirmed by obtaining the calibration curve for the H148G/V224L mutant. The constants were the following: *K*_*d*_ = 21.4 ± 4.8 mM, A1 = 1.58, A2 = 1.06, and *p* = 1.51 (Figure 6D). Statistical analysis of *K*_*d*_ values for ClopHensor vs. H148G/V224L showed that they were significantly different (*P* < 0.05, *t*-Student) and for PalmPalm vs. H148G/V224L *K*_*d*_ values were also significantly different (*P* < 0.01, *t*-Student). The dynamic range of *R*_*Cl*_ changes for the mutant was, however, smaller than those for the ClopHensor, which reduces the benefits arising from the increased affinity.

#### Calibration for pH

As the H148G/V224L mutation caused a shift in the p*K*_*a*_, we performed comparative pH calibrations of ClopHensor and H148G/V224L mutant. For pH calibration, the β-escin method was used (see Materials and Methods).

**Figure 7:**
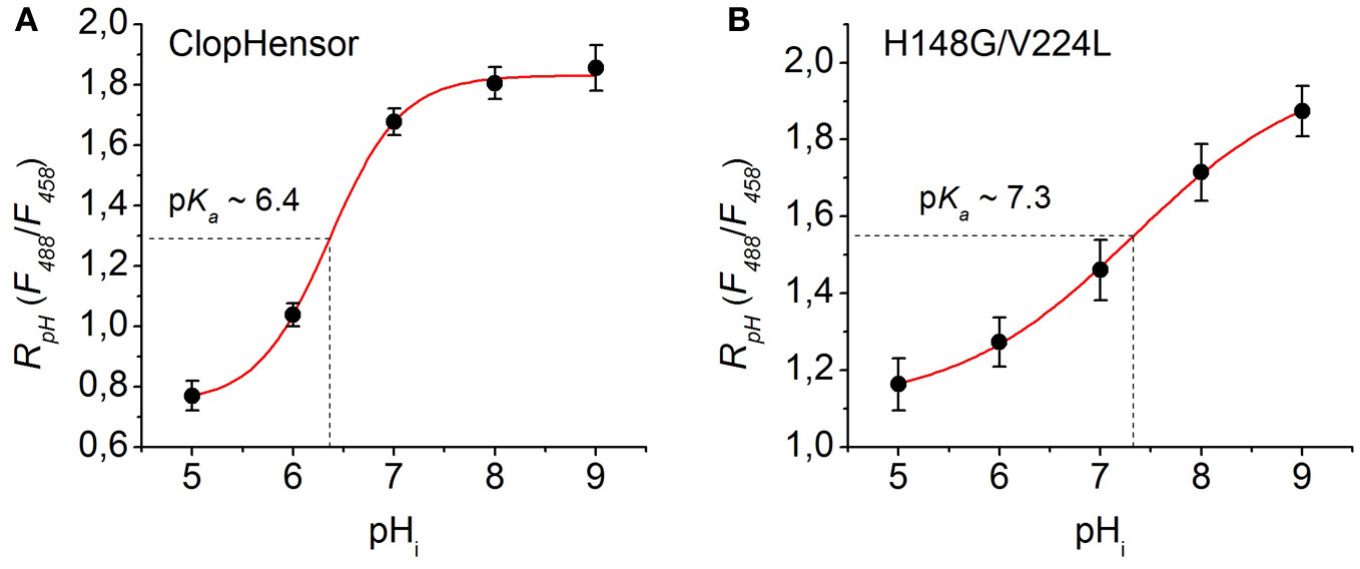
**Calibration of ClopHensor and H148G/V224L mutant for pH. (A)** and **(B)** Changes in *R*_*pH*_ (*F*_488_/*F*_458_) of ClopHensor **(A)** and H148G/V224L mutant **(B)** measured in transfected partially permeabilized CHO cells perfused with bath medium of different pH values (see Materials and Methods for details).

Sequential exchanging of bath solutions with different pH values produced corresponding shifts in the fluorescence pH ratio (Figure 7).

For pH calibration curves a Dose–Response Sigmoidal Fit (OriginPro 8.5) with the following formula was used:

$$R_{\text{pH}}=B1+\frac{B2-B1}{1+10^{\text{p}(\text{p}K_{a}-{\text{pH}}_{\text{i}})}}$$

where *R*_*pH*_ is the fluorescence ratio for pH (*F*_488_/*F*_458_), p*K*_*a*_ is the acid dissociation constant for H^+^ binding, B1 and B2 are, respectively, the minimum and maximum asymptotic values of *R*_*pH*_, and *p* is the power value.

From this formula we obtained the equation for pH_*i*_ values recalculation:

$${\text{pH}}_{\text{i}}=\text{p}K_{a}-\frac{1}{\text{p}}\cdot \log\left(\frac{B2-B1}{R_{\text{pH}}-B1}-1\right)$$

Based on the fitting of the experimental curves the constants values obtained were: p*K*_*a*_ = 6.36, B1 = 0.75, B2 = 1.83, and *p* = 1.21 for ClopHensor (Figure 7A) and p*K*_*a*_ = 7.32, B1 = 1.11, B2 = 1.99, and *p* = 0.49 for the H148G/V224L mutant (Figure 7B). In comparison with ClopHensor, the dynamic range of *R*_*pH*_ changes and the slope of the calibration curve characterizing by the constant *p* for the mutant were smaller, indicating its lower pH sensitivity.

#### Transients of [Cl^−^]_*i*_ and pH_*i*_ during activation glycine receptor channels

We further performed comparative analysis of the three variants of ClopHensor while monitoring [Cl^−^]_*i*_ and pH_*i*_ transients induced by activation of Cl^−^-selective GlyR channels in CHO cells.

**Figure 8:**
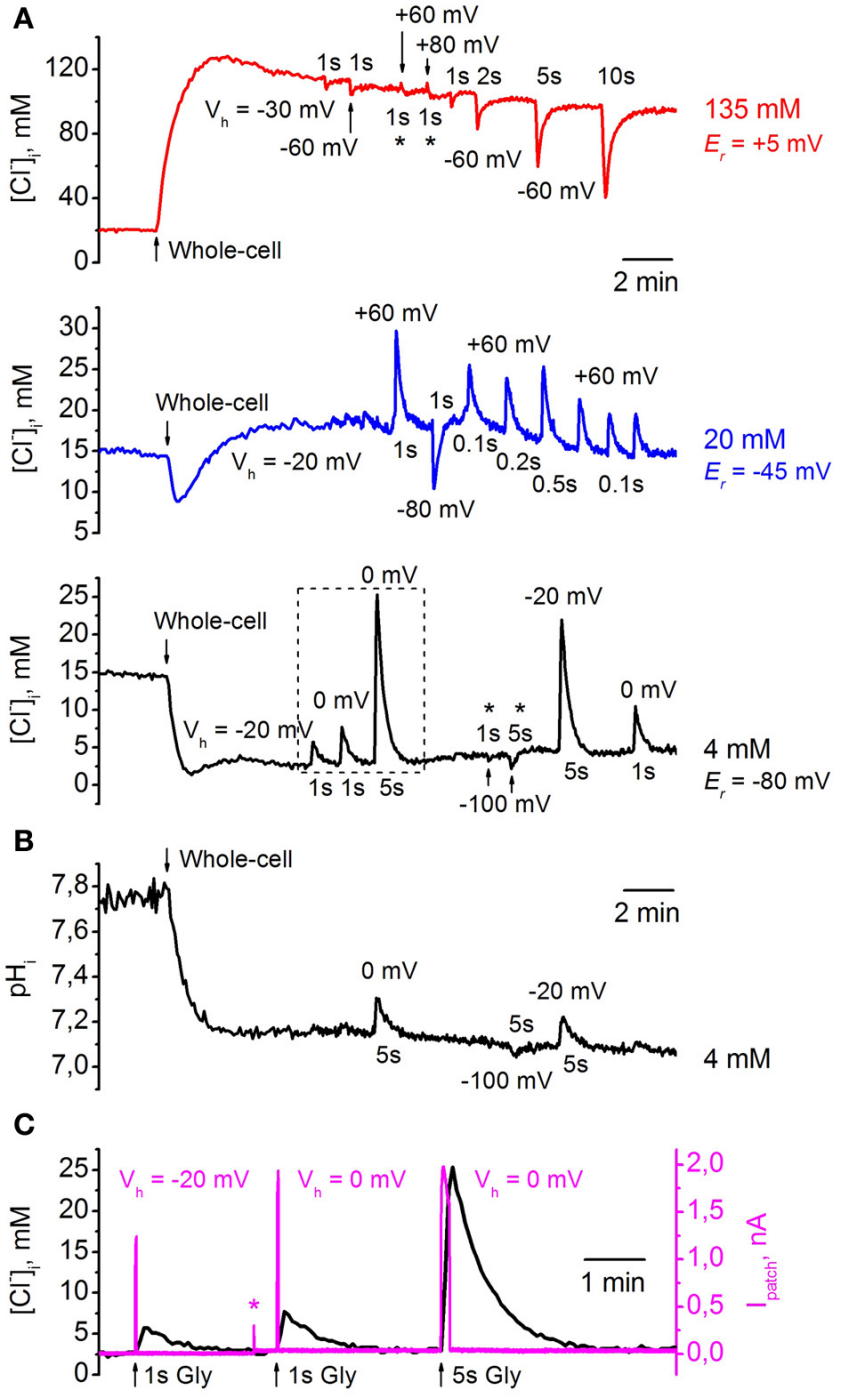
**Monitoring of transients of [Cl^−^]_*i*_ and pH_*i*_ during GlyR activation using ClopHensor. (A)** Changes in [Cl^−^]_*i*_ monitored by ClopHensor in three CHO cells co-expressing GlyR and recorded with different [Cl^−^]_*p*_: 4 mM (*black trace*), 20 mM (*blue trace*), and 135 mM (*red trace*). Transients in [Cl^−^]_*i*_ on transition to whole-cell configuration and GlyR channel activation at different holding potentials (*V*_*h*_) and different durations of glycine (200 μM) pulses are illustrated. Reversal potentials for glycine-induced currents (*E*_*r*_) for each cell recorded with different [Cl^−^]_*p*_ are indicated. **(B)** Changes in pH_*i*_ monitored simultaneously with [Cl^−^]_*i*_ by ClopHensor in the same cell as in **(A)** (*black trace*) recorded with [Cl^−^]_*p*_ = 4 mM. Note that only the long (5 s) glycine application produced noticeable pH_*i*_ changes of 0.1–0.2 units. **(C)** Simultaneous monitoring of whole-cell current (I_patch_) and [Cl^−^]_*i*_ during glycine application in a cell co-expressing ClopHensor and GlyR and recorded with a [Cl^−^]_*p*_ of 4 mM. Responses from *dashed frame* in **(A)** (*black trace*) are illustrated. Note: (i) the kinetics of [Cl^−^]_*i*_ transients are much slower than those for corresponding glycine-induced currents (I_Gly_); (ii) both amplitudes of I_Gly_ and [Cl^−^]_*i*_ changes increase with elevation of the driving force for Cl^−^ (recordings at *V*_*h*_ = −20 and 0 mV); (iii) [Cl^−^]_*i*_ amplitude increases with increasing GlyR channels activation from 1 to 5 s, while I_Gly_ amplitudes are similar (*V*_*h*_ = 0 mV). *Asterisk* on I_patch_ trace indicates the artifact when *V*_*h*_ changed from −20 to 0 mV. Because of the high input resistance of the cell, the steady-state shift in the current on changing the *V*_*h*_ is very weak in comparison with I_Gly_ amplitudes and is not visible on the record.

Figure 8A illustrates changes in [Cl^−^]_*i*_ monitored from three CHO cells co-expressing ClopHensor with GlyR and recorded with different [Cl^−^]_*p*_: 4 mM (*black trace*), 20 mM (*blue trace*), and 135 mM (*red trace*). Values of [Cl^−^]_*i*_ recalculated from *R*_*Cl*_ (*F*_458_/*F*_545_) were obtained using the calibration curve in Figure 5D. The transition to whole-cell configuration (holding potential *V*_*h*_ = −30 mV) with a pipette containing 135 mM Cl^−^ caused a strong elevation of [Cl^−^]_*i*_. After peaking, [Cl^−^]_*i*_ slowly declined, presumably due to pumping out of Cl^−^_*i*_ by transporters (see, for instance, Pellegrino et al., 2011) or through weakly activated Cl^−^-selective ionic channels (Friedel et al., 2013). Application of 200 μM glycine to the cell induced a transient decrease in [Cl^−^]_*i*_, the amplitude of which depended on the duration of the applied pulses of the agonist and values of *V*_*h*_. The holding potential was transiently changed to the values indicated on the figure for each pulse of glycine. Application of the agonist for 1 s caused a decrease of 5–10 mM, while activation of GlyRs for 10 s resulted in decrease of about 60 mM (Figure 8A, *red trace*). After transiently diminishing, [Cl^−^]_*i*_ recovered to its initial steady-state level. Interestingly, for the cell recorded with [Cl^−^]_*p*_ = 135 mM it was difficult to increase [Cl^−^]_*i*_ in conditions under which the glycine-induced current caused a strong influx of Cl^−^, i.e., at *V*_*h*_ = +60 and even at +80 mV (Figure 8A, *asterisks*, *red trace*). In this cell the reversal potential (*E*_*r*_) for I_Gly_ was about +5 mV, as predicted for symmetric intracellular–extracellular Cl^−^ concentrations.

Simultaneous recording of [Cl^−^]_*i*_ and ionic currents (Figure 8C) showed two main features. First, the kinetics of [Cl^−^]_*i*_ transients were much slower than those for glycine-induced currents. This is in accordance with previous observations from monitoring of Cl^−^ transients using MQAE-mediated fluorescence (Marandi et al., 2002) or BioSensor-GlyR (Mukhtarov et al., 2008). Second, [Cl^−^]_*i*_ amplitude increased both on elevation of the driving force for Cl^−^ and on prolongation of glycine applications. As illustrated in Figure 8C, the amplitude of [Cl^−^]_*i*_ transients induced by 1-s pulses of glycine increased in parallel with GlyR current amplitude at changes of *V*_*h*_ from −20 to 0 mV. At constant *V*_*h*_ (0 mV), prolongation of glycine pulses from 1 to 5 s caused an increase in [Cl^−^]_*i*_ amplitude although an increase in GlyR current amplitude was not observed.

When transition to whole-cell configuration (*V*_*h*_ = −20 mV) was produced with a pipette containing 20 mM Cl^−^ a brief transient decrease in [Cl^−^]_*i*_ with subsequent stabilization at a level close to that in the cell-attached configuration was observed (Figure 8A, *blue trace*). Even at short (100 ms) glycine applications, substantial transients in [Cl^−^]_*i*_, of about 5 mM, were recorded with *V*_*h*_ = +60 mV; these reversed in direction when *V*_*h*_ was changed from +60 to −80 mV. *E*_*r*_ for glycine-induced currents in this cell was about −45 mV (Figure 8A, *blue trace*).

With a pipette containing 4 mM Cl^−^, transition to whole-cell configuration (*V*_*h*_ = −20 mV), as predicted, caused a decrease in [Cl^−^]_*i*_ (Figure 8A, *black trace*). At *V*_*h*_ = −20 and 0 mV, application of 200 μM glycine induced strong [Cl^−^]_*i*_ transients whose amplitudes increased with prolongation of pulse duration; these increases were about 3–5 and 18–22 mM for glycine pulses of 1 and 5 s, respectively. Similarly to high [Cl^−^]_*p*_, for the cell recorded with a [Cl^−^]_*p*_ of 4 mM it was difficult to decrease [Cl^−^]_*i*_ in conditions under which glycine-induced current caused efflux of Cl^−^, i.e., at *V*_*h*_ = −100 mV (Figure 8A, *asterisks*, *black trace*). *E*_*r*_ for glycine-induced currents in this cell was about −80 mV.

Simultaneous monitoring of pH_*i*_ in the same cell as that shown in Figure 8A, *black trace*, demonstrated a decrease in pH_*i*_ of about 0.6 units after transition to whole-cell configuration (Figure 8B). Values of pH_*i*_ recalculated from *R*_*pH*_ (*F*_488_/*F*_458_) were obtained using the calibration curve in Figure 7A. The changes in [Cl^−^]_*i*_ up to 5 mM (with 1-s glycine applications) did not produce marked changes in pH_*i*_, while the changes in [Cl^−^]_*i*_ to ≥20 mM (with 5-s glycine applications) caused changes in pH_*i*_ of 0.1–0.2 units (Figure 8B).

These observations indicate that ClopHensor is a reliable tool for long-lasting simultaneous monitoring of intracellular Cl^−^ and pH in living cells.

Next we analysed the properties of ClopHensor derivatives: the membrane-targeted PalmPalm-ClopHensor and the H148G/V224L ClopHensor mutant.

**Figure 9:**
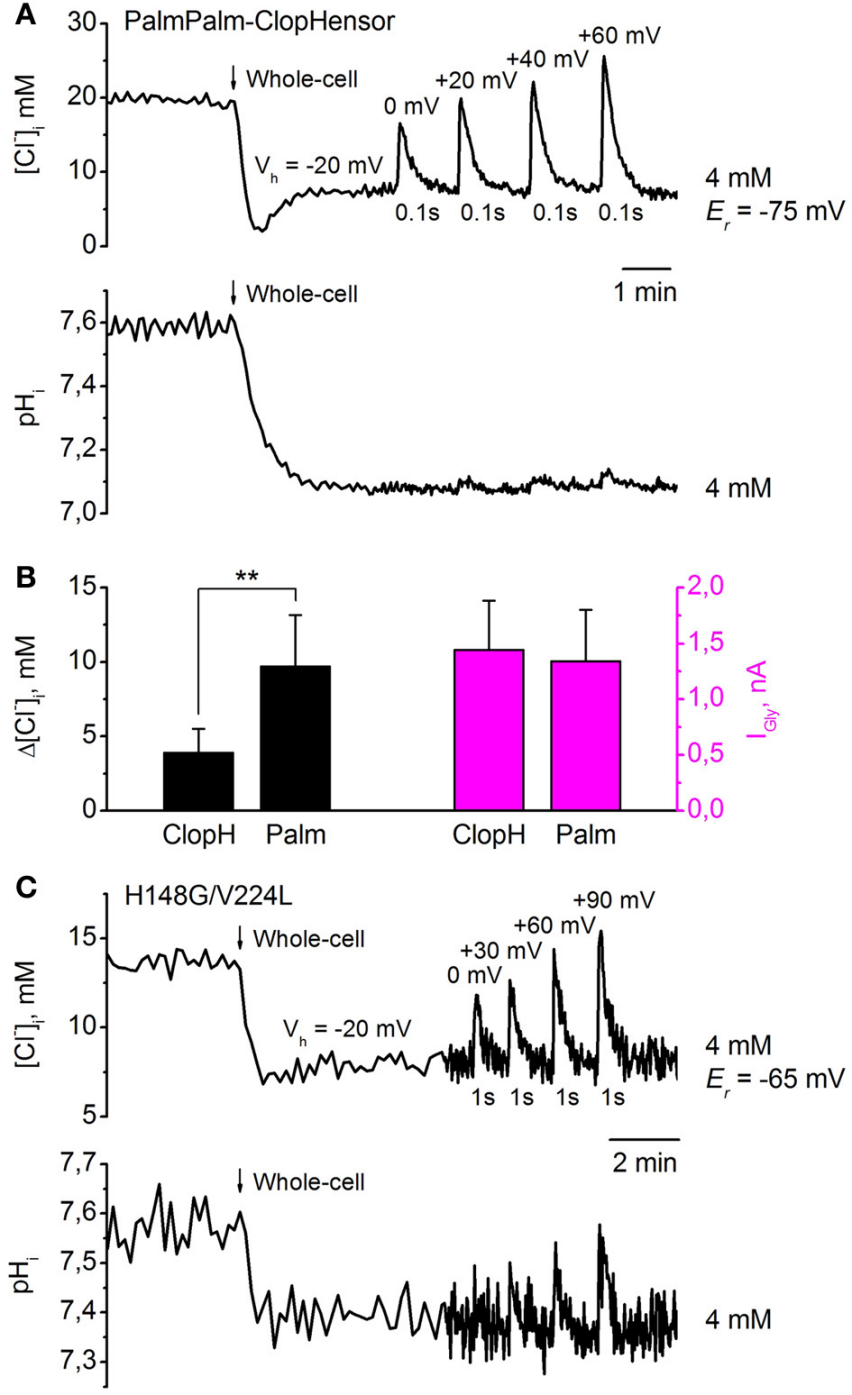
**Monitoring of transients in [Cl^−^]_*i*_ and pH_*i*_ in response to GlyR activation, using PalmPalm-ClopHensor and the H148G/V224L mutant. (A)** Changes in [Cl^−^]_*i*_ (*top trace*) and pH_*i*_ (*bottom trace*) monitored simultaneously by PalmPalm-ClopHensor in a CHO cell co-expressing GlyR and recorded with [Cl^−^]_*p*_ = 4 mM. Transients in [Cl^−^]_*i*_ and pH_*i*_ upon whole-cell penetration and GlyR activation by pressure application of 200 μM glycine to the cell at different *V*_*h*_ and 100 ms duration of agonist application are illustrated. The reversal potential for glycine-induced currents (*E*_*r*_) is indicated. Note the very small pH_*i*_ changes at short (100 ms) glycine application (*bottom trace*). **(B)** Averaged glycine-induced current (I_Gly_) amplitudes (*magenta columns, right*) and corresponding [Cl^−^]_*i*_ changes (*black columns, left*) in response to 1-s glycine applications. Whole-cell recordings from CHO cells with pipettes containing [Cl^−^]_*p*_ = 4 mM. Cells expressed either ClopHensor (*ClopH*) or PalmPalm-ClopHensor (Palm). Note that for very similar I_Gly_, transients of [Cl^−^]_*i*_ are about 2.5-fold higher when monitored by PalmPalm-ClopHensor than by ClopHensor. Data from seven cells for each sensor are illustrated. ^**^P < 0.01 (ANOVA). **(C)** Changes in [Cl^−^]_*i*_ (*top trace*) and pH_*i*_ (*bottom trace*) monitored by the H148G/V224L mutant of ClopHensor in a CHO cell co-expressing GlyR and recorded with a [Cl^−^]_*p*_ of 4 mM. Transients in [Cl^−^]_*i*_ upon whole-cell penetration and GlyR activation by pressure application of 200 μM glycine to the cell at different V_*h*_ and 1-s duration of agonist application are illustrated. Note the evident pH_*i*_ changes of about 0.1 units only under conditions of substantial difference between *V*_*h*_ and *E*_*r*_ and relatively long (1-s) glycine application. Different baseline noise in the two traces resulted from changes in acquisition frequency from 0.2 to 1 Hz.

Figure 9A illustrates simultaneous monitoring of [Cl^−^]_*i*_ (*top trace*) and pH_*i*_ (*bottom trace*) by PalmPalm-ClopHensor in CHO cells co-expressing GlyR and recorded with a [Cl^−^]_*p*_ of 4 mM. Values of [Cl^−^]_*i*_ recalculated from *R*_*Cl*_ (*F*_545_/*F*_458_) were obtained using the calibration curve in Figure 6B. The transition to whole-cell configuration (*V*_*h*_ = −20 mV) produced a decrease in [Cl^−^]_*i*_ similar to those monitored by ClopHensor. Short (100 ms) application of 200 μM glycine to the cell induced transient increases in [Cl^−^]_*i*_ that were dependent on the difference between the holding potential and the reversal potential of glycine-induced currents.

Transients in [Cl^−^]_*i*_ varied from about 10 mM at *V*_*h*_ = 0 mV to about 20 mM at *V*_*h*_ = +60 mV (Figure 9A, *top trace*). *E*_*r*_ for glycine-induced currents in this cell was about −75 mV. These changes were much higher than would be found when monitoring with ClopHensor at similar stimuli. This was confirmed on analysis of [Cl^−^]_*i*_ transients when recording from cells expressing these two sensors. As illustrated in Figure 9B, very similar GlyR currents induced [Cl^−^]_*i*_ transients whose amplitude appeared about 2.5-fold higher when monitored by PalmPalm-ClopHensor.

As the molecular organization of PalmPalm-ClopHensor is identical to that of ClopHensor, the recalculation of pH_*i*_ values from *R*_*pH*_ (*F*_488_/*F*_458_) was done using the calibration curve in Figure 7A. Similarly, after transition to whole-cell configuration, pH_*i*_ decreased by about 0.5 units (Figure 9A, *bottom trace*). Very weak pH_*i*_ transients were observed (Figure 9A, *bottom trace*) with short (100 ms) glycine applications even at *V*_*h*_ = +60 mV, while the transient increase in [Cl^−^]_*i*_ in this cell was about 20 mM (Figure 9A, *top trace*).

When the H148G/V224L mutant of ClopHensor was co-expressed with GlyR in CHO cells recorded with [Cl^−^]_*p*_ = 4 mM, a decrease in [Cl^−^]_*i*_ on transition to whole-cell configuration (*V*_*h*_ = −20 mV) was also observed (Figure 9C, *top trace*). Values of [Cl^−^]_*i*_ recalculated from *R*_*Cl*_(*F*_545_/*F*_458_) were obtained using the calibration curve in Figure 6D. Again, glycine-induced transient increases in [Cl^−^]_*i*_ depended on the difference between V_*h*_ and *E*_*r*_. Transients in [Cl^−^]_*i*_ with 1-s glycine application varied from about 5 mM at *V*_*h*_ = 0 mV to about 10 mM at *V*_*h*_ = +90 mV. *E*_*r*_ for glycine-induced currents in this cell was about −65 mV.

Simultaneous monitoring of pH_*i*_ in the same cell reveal that the [Cl^−^]_*i*_ transients below 5 mM did not produce marked changes in pH_*i*_. The recalculation of pH_*i*_ values from *R*_*pH*_(*F*_488_/*F*_458_) was done using the calibration curve in Figure 7B. An evident pH_*i*_ increase (of about 0.1 units) was recorded only when there was a substantial difference between *V*_*h*_ and *E*_*r*_ and relatively long (1-s) glycine application, which induced a [Cl^−^]_*i*_ elevation of about 10 mM (Figure 9C, *bottom trace*).

These results demonstrate that PalmPalm-ClopHensor, which has preferable membrane targeting, could be of interest for near-membrane measurement of intracellular Cl^−^ and pH changes. Another construct, the H148G/V224L mutant of ClopHensor, with a higher affinity for Cl^−^, possesses the smallest dynamic range of *R*_*Cl*_ (*F*_458_/*F*_545_) changes and this could be a limiting factor in some cases due to lower signal/noise ratio.

## Discussion

Development of genetically encoded probes for non-invasive monitoring of ions and protein function has opened powerful routes for the analysis of a variety of physiological problems and functions of various cell types under different experimental conditions. These probes are non-toxic, capable of remaining stable in cells for a long time, can be expressed in specific cellular compartments and are suitable for production of transgenic models.

Here, we describe the calibration in living cells, the cytoplasmic distribution, and examples of simultaneous monitoring of intracellular Cl^−^ and H^+^ of three genetically encoded sensors: (1) ClopHensor; (2) its variant designed to have preferential membrane targeting due to the addition to the N-terminus of a short peptide containing two palmitoylation sites (PalmPalm-ClopHensor) (Arosio et al., 2010), and (3) ClopHensor containing mutations of E^2^GFP (H148G/V224L) aimed at increasing the Cl^−^ affinity of the sensor.

Following transient expression in CHO cells, ClopHensor and the H148G/V224L mutant exhibit cytoplasmic intracellular distribution while the PalmPalm-ClopHensor construct, as expected, is preferentially localized in the vicinity of membranes. Upon transfection of rat dissociated hippocampal cultures with cDNA of ClopHensor, the probe shows a strong cytoplasmically distributed fluorescence in the soma and neuronal processes. These observations indicate that all three sensors can be easily expressed in various cell types and detected in different, even very small, areas of cells.

Calibration analysis performed on CHO cells using patch pipettes containing different concentrations of Cl^−^, and also β-escin permeabilization with bath medium having different pH (Waseem et al., 2010) revealed that the constructs exhibit different sensitivity to Cl^−^ and H^+^. While ClopHensor and PalmPalm-ClopHensor probes demonstrated *K*_*d*_ for Cl^−^ of about 40 mM, for the H148G/V224L mutant this value was about 20 mM, indicating its higher affinity. However, the mutant exhibits a smaller dynamic range of *R*_*Cl*_, which could be a limiting factor for monitoring Cl^−^ in cells containing low [Cl^−^]_*i*_ or with small changes in Cl^−^ concentration. Poor dynamic range could be, at least partially, explained by different spectral properties of H148G/V224L mutant (Figures 2A,C) from those for ClopHensor (Arosio et al., 2010). In the present study, for all constructs including the H148G/V224L mutant, the calibration measurements were performed using the same excitation wavelengths, GFP/DsRed dichroic mirror and emission filters (see Materials and Methods). We suggest that this range can be considerably extended in the future by better selection of excitation and emission parameters.

Calibration of pH in CHO cells using the β-escin method showed that the p*K*_*a*_ for the H148G/V224L mutant is strongly shifted to alkaline values. This is consistent with *in vitro* measurements: p*K*_*a*_ = 7.9 ± 0.05. *In vitro* analysis also demonstrated that *K*_*d*_ for Cl^−^ of the H148G/V224L mutant exhibits relatively small pH dependency over a wide pH range: from about 18 mM at pH 6.5 to about 30 mM at pH 7.8. This suggests that the H148G/V224L mutant is a useful tool for [Cl^−^]_*i*_ measurements in experimental models with high pH variations.

Our previous observations demonstrated that when using the CFP/YFP-based Cl-Sensor, long or frequent acquisition causes strong “bleaching” of Cl^−^-sensitive YFP resulting in changes in fluorescence parameters during ratiometric measurement of [Cl^−^]_*i*_. This problem is discussed in detail in the paper by Friedel et al. (2013, this issue). In contrast, E^2^GFP-DsRedm-based ClopHsensor exhibits remarkable stability (see, for instance, Figures 8, 9), providing an excellent tool for long-lasting reliable monitoring and with variable acquisition rate.

Our observations of the effects of activation of Cl^−^-selective GlyR channels on [Cl^−^]_*i*_ indicate that it is a highly dynamic parameter, which can be strongly changed by overactivation of Cl^−^-selective channels or activity of other proteins involved in regulation and determination of physiological [Cl^−^]_*i*_ in living systems. Indeed, activation of GlyRs for several seconds caused changes in [Cl^−^]_*i*_ of more than 10–20 mM (Figures 8, 9).

Together our experiments demonstrate that these three ClopHensor constructs are suitable tools for stable, long-lasting, non-invasive monitoring of [Cl^−^]_*i*_ and pH_*i*_ in different cell types.

### Conflict of interest statement

The authors declare that the research was conducted in the absence of any commercial or financial relationships that could be construed as a potential conflict of interest.

## References

[B1] Arosio D. Beltram F. Ricci F. Marchetti L. (2011). Novel pH- and anion concentration-responsive GFP mutant, a chimeric protein comprising such a mutant and a method for the combined assaying of the pH and anion concentration. Eur. Pat. Appl. 08165 522.7–1212.

[B2] Arosio D. Garau G. Ricci F. Marchetti L. Bizzarri R. Nifosì R. (2007). Spectroscopic and structural study of proton and halide ion cooperative binding to gfp. Biophys. J. 93, 232–244 10.1529/biophysj.106.102319 17434942 PMC1914440

[B3] Arosio D. Ricci F. Marchetti L. Gualdani R. Albertazzi L. Beltram F. (2010) Simultaneous intracellular chloride and pH measurements using a GFP-based sensor. Nat. Methods 7, 516–518 10.1038/nmeth.1471 20581829

[B4] Batti L. Mukhtarov M. Audero E. Ivanov A. Paolicelli R. Zurborg S. (2013). Transgenic mouse lines for non-invasive ratiometric monitoring of intracellular chloride. Front. Mol. Neurosci. 10.3389/fnmol.2013.00011 PMC3659292 23734096

[B5] Berglund K. Kuner T. Feng G. Augustine G. J. (2011). Imaging synaptic inhibition with the genetically encoded chloride indicator Clomeleon. Cold Spring Harb. Protoc. 2011, 1492–1497 10.1101/pdb.prot066985 22135666

[B6] Berglund K. Schleich W. Wang H. Feng G. Hall W. C. Kuner T. (2008). Imaging synaptic inhibition throughout the brain via genetically targeted Clomeleon. Brain Cell Biol. 36, 101–118 10.1007/s11068-008-9031-x 18850274 PMC2674236

[B7] Bertollini C. Murana E. Mosca L. D'Erme M. Scala F. Francioso A. (2012) Transient increase in neuronal chloride concentration by neuroactive aminoacids released from glioma cells. Front. Mol. Neurosci. 5:100 10.3389/fnmol.2012.00100 PMC3505843 23189038

[B8] Bregestovski P. Arosio D. (2011). Green fluorescent protein-based chloride ion sensors for *in vivo* imaging, in Fluorescent Proteins, Springer Ser Fluoresc, ed Jung G. (Berlin, Heidelberg: Springer-Verlag), 90–124

[B9] Bregestovski P. Waseem T. Mukhtarov M. (2009). Genetically encoded optical sensors for monitoring of intracellular chloride and chloride-selective channel activity. Front. Mol. Neurosci. 2:15 10.3389/neuro.02.015.2009 20057911 PMC2802328

[B10] Buerli T. Pellegrino C. Baer K. Lardi-Studler B. Chudotvorova I. Fritschy J. M. (2007). Efficient transfection of DNA or shRNA vectors into neurons using magnetofection. Nat. Protoc. 2, 3090–3101 10.1038/nprot.2007.445 18079708

[B11] Dzhala V. Valeeva G. Glykys J. Khazipov R. Staley K. (2012). Traumatic alterations in GABA signaling disrupt hippocampal network activity in the developing brain. J. Neurosci. 32, 4017–4031 10.1523/JNEUROSCI.5139-11.2012 22442068 PMC3333790

[B12] Elsliger M. A. Wachter R. M. Hanson G. T. Kallio K. Remington S. J. (1999) Structural and spectral response of green fluorescent protein variants to changes in pH. Biochemistry 38, 5296–5301 10.1021/bi9902182 10220315

[B13] Friedel P. Bregestovski P. Medina I. (2013). Improved method for efficient imaging of intracellular Cl^−^ with Cl^−^ Sensor using conventional fluorescence set up. Front. Mol. Neurosci. 6:7 10.3389/fnmol.2013.00007 PMC3622059 23596389

[B14] Hanson G. T. McAnaney T. B. Park E. S. Rendell M. E. Yarbrough D. K. Chu S. (2002). Green fluorescent protein variants as ratiometric dual emission pH sensors. 1. Structural characterization and preliminary application. Biochemistry 41, 15477–15488 12501176 10.1021/bi026609p

[B15] Khirug S. Yamada J. Afzalov R. Voipio J. Khiroug L. Kaila K. (2008). GABAergic depolarization of the axon initial segment in cortical principal neurons is caused by the Na-K-2Cl cotransporter NKCC1. J. Neurosci. 28, 4635–4639 10.1523/JNEUROSCI.0908-08.2008 18448640 PMC6670448

[B16] Kuner T. Augustine G. J. (2000). A genetically encoded ratiometric indicator for chloride: capturing chloride transients in cultured hippocampal neurons. Neuron 27, 447–459 10.1016/S0896-6273(00)00056-8 11055428

[B17] Mancuso J. J. Kim J. Lee S. Tsuda S. Chow N. B. Augustine G. J. (2011). Optogenetic probing of functional brain circuitry. Exp. Physiol. 96, 26–33 10.1113/expphysiol.2010.055731 21056968

[B18] Marandi N. Konnerth A. Garaschuk O. (2002). Two-photon chloride imaging in neurons of brain slices. Pflugers Arch. 445, 357–365 10.1007/s00424-002-0933-7 12466938

[B19] Markova O. Mukhtarov M. Real E. Jacob Y. Bregestovski P. (2008). Genetically encoded chloride indicator with improved sensitivity. J. Neurosci. Methods 170, 67–76 10.1016/j.jneumeth.2007.12.016 18279971

[B20] McCabe J. B. Berthiaume L. G. (1999). Functional roles for fatty acylated amino-terminal domains in subcellular localization. Mol. Biol. Cell 10, 3771–3786 10564270 10.1091/mbc.10.11.3771 PMC25678

[B20a] Medina I. Krapivinsky G. Arnold S. Kovoor P. Krapivinsky L. Clapham D. E. (2000). A switch mechanism for G beta gamma activation of I(KACh). J. Biol. Chem. 275, 29709–29716 10.1074/jbc.M004989200 10900209

[B21] Mukhtarov M. Markova O. Real E. Jacob Y. Buldakova S. Bregestovski P. (2008). Monitoring of chloride and activity of glycine receptor channels using genetically encoded fluorescent sensors. Philos. Transact. A Math. Phys. Eng. Sci. 366, 3445–3462 10.1098/rsta.2008.0133 18632458

[B22] Pellegrino C. Gubkina O. Schaefer M. Becq H. Ludwig A. Mukhtarov M. (2011) Knocking down of the KCC2 in rat hippocampal neurons increases intracellular chloride concentration and compromises neuronal survival. J. Physiol. 589, 2475–2496 10.1113/jphysiol.2010.203703 21486764 PMC3115820

[B23] Raimondo J. V. Irkle A. Wefelmeyer W. Newey S. E. Akerman C. J. (2012). Genetically encoded proton sensors reveal activity-dependent pH changes in neurons. Front. Mol. Neurosci. 5:68 10.3389/fnmol.2012.00068 22666186 PMC3364509

[B24] Tsien R. Y. Pozzan T. Rink T. J. (1982) Calcium homeostasis in intact lymphocytes: cytoplasmic free calcium monitored with a new, intracellularly trapped fluorescent indicator. J. Cell Biol. 94, 325–334 6980885 10.1083/jcb.94.2.325 PMC2112871

[B25] Tyzio R. Minlebaev M. Rheims S. Ivanov A. Jorquera I. Holmes G. L. (2008). Postnatal changes in somatic gamma-aminobutyric acid signalling in the rat hippocampus. Eur. J. Neurosci. 27, 2515–2528 10.1111/j.1460-9568.2008.06234.x 18547241

[B26] Waseem T. Mukhtarov M. Buldakova S. Medina I. Bregestovski P. (2010). Genetically encoded Cl-Sensor as a tool for monitoring of Cl-dependent processes in small neuronal compartments. J. Neurosci. Methods 193, 14–23 10.1016/j.jneumeth.2010.08.002 20705097

